# Supplementary material for: Impacts of Thermal Processing, High Pressure, and CO2-Assisted High Pressure on Quality Characteristics and Shelf Life of Durian Fruit Puree
Source: Foods. 2022 Sep 5;11(17):2717. doi: 10.3390/foods11172717 (PMC9455942; doi:10.3390/foods11172717)
Supplement: Supplementary file 1 [file foods-11-02717-s001.zip › foods-1876393-supplementary.pdf]

# Supplementary materials

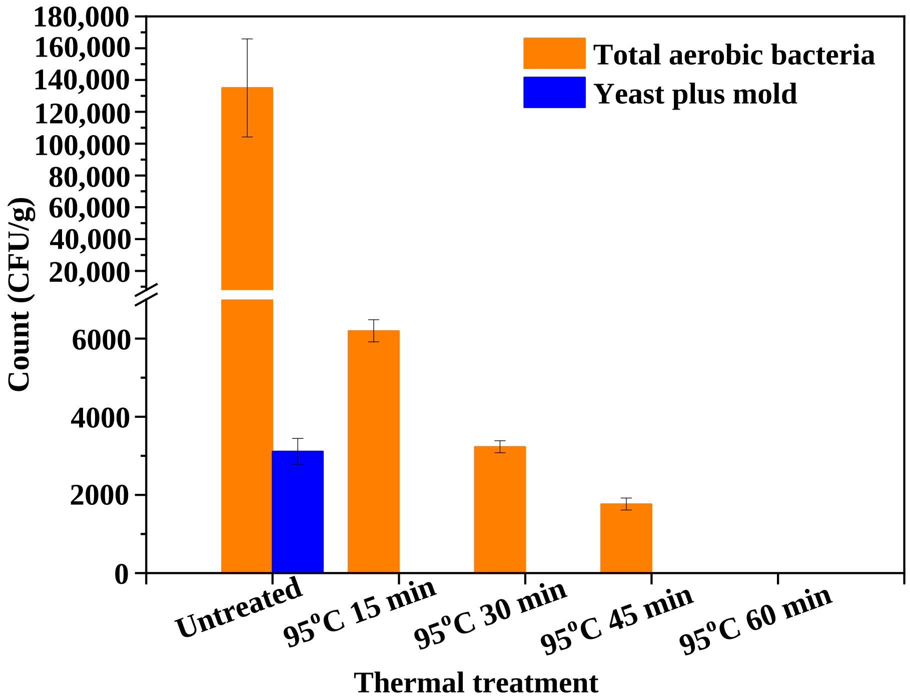


**Figure S1 Effects of TP treatment with different treated time on total aerobic bacteria and yeast plus mold counts of DFP.**

**Table S1 K value and n value of different DFP samples**

| Treatments | Storage time (days) | | | | | |
|---|---|---|---|---|---|---|
| | 0 | 7 | 14 | 21 | 28 | 35 |
| Untreated (K) | 3.2±0.21 | - | - | - | - | - |
| TP (K) | 4.96±0.51 | 6.54±1.33 | 8.13±1.56 | 6.82±1.29 | 12.88±1.56 | 14.54±2.16 |
| HPP (K) | 3.85±0.49 | 6.58±1.11 | 9.58±2.01 | 8.13±1.56 | 7.01±1.36 | 8.84±1.1 |
| $CO_2$ + HPP (K) | 1.42±0.17 | 1.36±0.05 | 3.56±0.38 | 7.07±1.29 | 7.98±1.24 | 6.23±0.63 |
| Untreated (n) | 0±0.01 | - | - | - | - | - |
| TP (n) | 0.14±0.02 | 0.17±0.05 | 0.2±0.05 | 0.13±0.04 | 0.14±0.03 | 0.13±0.03 |
| HPP (n) | 0.16±0.03 | 0.26±0.04 | 0.28±0.05 | 0.2±0.05 | 0.12±0.05 | 0.11±0.03 |
| $CO_2$ + HPP (n) | 0.01±0.03 | -0.05±0.01 | 0.12±0.02 | 0.21±0.04 | 0.21±0.04 | 0.14±0.02 |
